# Supplementary material for: Inhibition of PI3K p110δ activity reduces IgE production in IL‐4 and anti‐CD40 stimulated human B cell cultures
Source: Immunology. 2023 Aug 2;170(4):483–94. doi: 10.1111/imm.13684 (PMC11495259; doi:10.1111/imm.13684)
Supplement: Supplementary file 1 — Supplementary Figure 1 (A) IL4 and anti‐CD40 stimulated cells were harvested from the day 10 cultures containing different concentrations of IC87114 and labelled with a live/dead fixable dye to determine the percentage of viable cells in culture. (B) The percentages of live cells made relative to the IL‐4 and anti‐CD40 only cultures [file IMM-170-483-s001.pdf]

Inhibition of PI3K p110δ activity reduces IgE production in IL-4 and anti-CD40 stimulated human B cell cultures

Anna Cutrina-Pons[1*], Aloka De Sa[1*], David J Fear[2], Hannah J Gould[1] and Faruk Ramadani[1]

Author Affiliations: [1] Randall Centre of Cell & Molecular Biophysics, and [2] Division of Asthma, Allergy and Lung Biology, King's College London, United Kingdom. *The first two authors contributed equally to this article, and both should be considered first author.

Correspondence: Faruk Ramadani, Randall Centre of Cell and Molecular Biophysics, King's College London, London, SE1 1UL, UK; e-mail: faruk.ramadani@kcl.ac.uk or Phone: 44 (0)20 7848 6442; Fax: +44 (0)20 7848 6435.

S1A

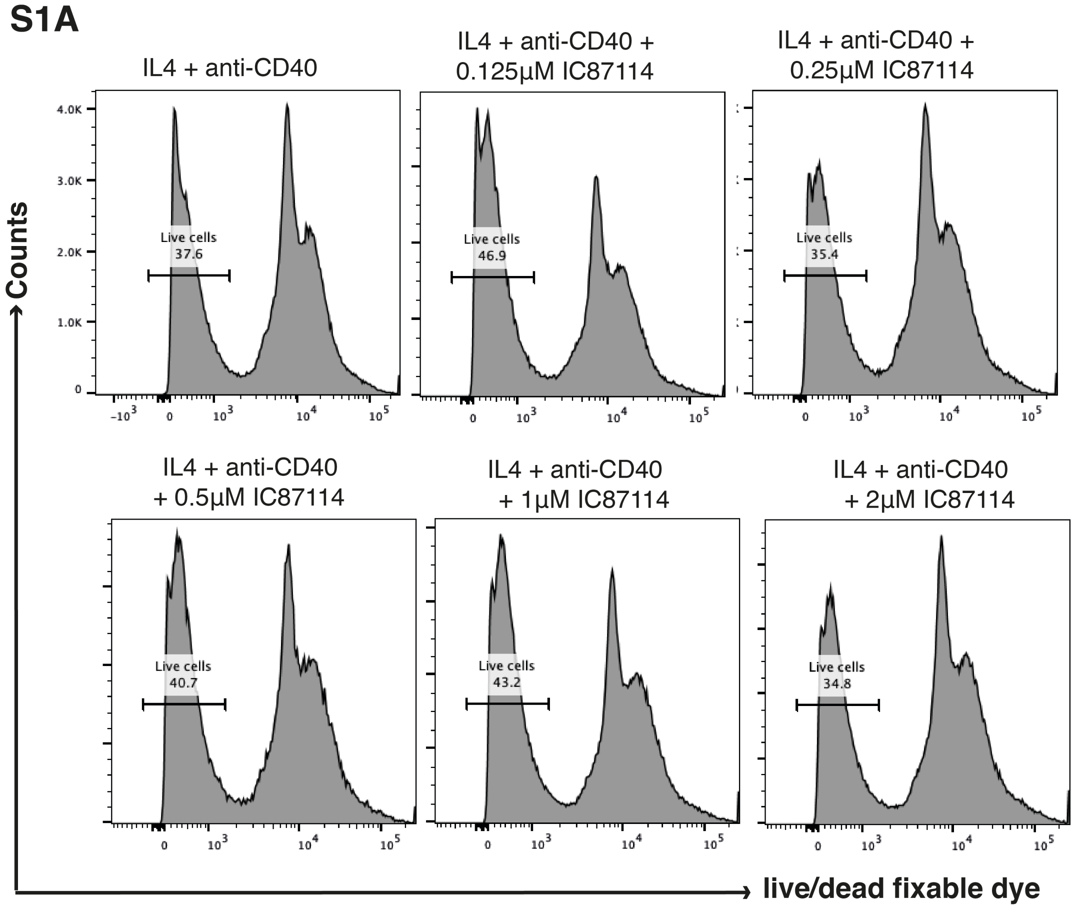

S1B

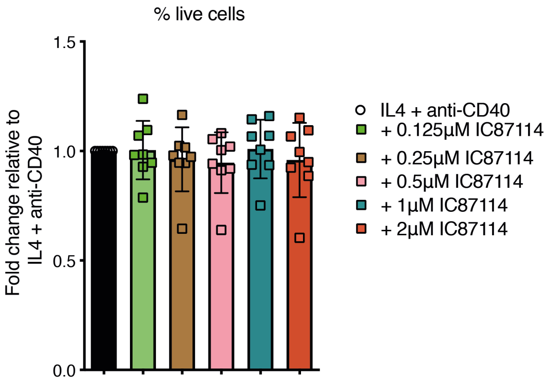

Supplementary Figure 1

S1A) IL4 and anti-CD40 stimulated cells were harvested from the day 10 cultures containing different concentrations of IC87114 and labelled with a live/dead fixable dye to determine the percentage of viable cells in culture.

S1B) The percentages of live cells made relative to the IL-4 and anti-CD40 only cultures
